# Comparing two strategies of dynamic intensity modulated radiation therapy (dIMRT) with 3-dimensional conformal radiation therapy (3DCRT) in the hypofractionated treatment of high-risk prostate cancer

**DOI:** 10.1186/1748-717X-3-1

**Published:** 2008-01-07

**Authors:** Jasper Yuen, George Rodrigues, Kristina Trenka, Terry Coad, Slav Yartsev, David D'Souza, Michael Lock, Glenn Bauman

**Affiliations:** 1 Department of Radiation Oncology, London Regional Cancer Program, London, Ontario, Canada; 2 Department of Epidemiology and Biostatistics, University of Western Ontario, London, Ontario, Canada; 3 Department of Clinical Physics, London Regional Cancer Program, London Health Sciences Centre, London, ON, Canada

## Abstract

**Background:**

To compare two strategies of dynamic intensity modulated radiation therapy (dIMRT) with 3-dimensional conformal radiation therapy (3DCRT) in the setting of hypofractionated high-risk prostate cancer treatment.

**Methods:**

3DCRT and dIMRT/Helical Tomotherapy(HT) planning with 10 CT datasets was undertaken to deliver 68 Gy in 25 fractions (prostate) and simultaneously delivering 45 Gy in 25 fractions (pelvic lymph node targets) in a single phase. The paradigms of pelvic vessel targeting (iliac vessels with margin are used to target pelvic nodes) and conformal normal tissue avoidance (treated soft tissues of the pelvis while limiting dose to identified pelvic critical structures) were assessed compared to 3DCRT controls. Both dIMRT/HT and 3DCRT solutions were compared to each other using repeated measures ANOVA and post-hoc paired t-tests.

**Results:**

When compared to conformal pelvic vessel targeting, conformal normal tissue avoidance delivered more homogenous PTV delivery (2/2 t-test comparisons; p < 0.001), similar nodal coverage (8/8 t-test comparisons; p = ns), higher and more homogenous pelvic tissue dose (6/6 t-test comparisons; p < 0.03), at the cost of slightly higher critical structure dose (D_dose_, 1–3 Gy over 5/10 dose points; p < 0.03). The dIMRT/HT approaches were superior to 3DCRT in sparing organs at risk (22/24 t-test comparisons; p < 0.05).

**Conclusion:**

dIMRT/HT nodal and pelvic targeting is superior to 3DCRT in dose delivery and critical structure sparing in the setting of hypofractionation for high-risk prostate cancer. The pelvic targeting paradigm is a potential solution to deliver highly conformal pelvic radiation treatment in the setting of nodal location uncertainty in prostate cancer and other pelvic malignancies.

## Background

Prostate cancer is the most common malignancy to afflict the Canadian male population. It is estimated that approximately 20700 men were diagnosed with prostate cancer in 2006 and approximately 4200 will die of this disease [1]. Standard curative treatment for high-risk prostate cancer [2] is a radical course of radiation treatment with long-term androgen suppression therapy [3,4]. A recently completed RTOG (Radiation Therapy Oncology Group) prospective randomized phase III trial shows that whole pelvic nodal irradiation improves biochemical disease-free survival in patients with a high-risk (>15%) of positive pelvic lymph nodes from prostate cancer based on tumour stage, PSA, and Gleason grade [5].

This radiation treatment usually consists of sequential phases using shrinking fields. Traditionally, the first phase consists of five daily fractions each week to the whole pelvis including the prostate gland and pelvic lymph nodes at risk using a four-field box technique. The usual prescribed doses range from 44 to 50.4 Gy in 1.8–2.0 Gy fractions. The remainder of the radiation treatment is given to a reduced boost volume targeting the prostate gland (± seminal vesicles) using the same fractionation schedule to a radical total dose. Androgen suppression therapy can be given in neo-adjuvant, concurrent, and/or adjuvant form with the radiation [3,4]. Unfortunately, the use of conventionally planned whole pelvic radiotherapy to treat the whole pelvis results in toxicity to normal structures such as the small bowel, rectum, and bladder.

Recent studies have illustrated a steep dose response relationship through escalating the total dose to approximately 80 Gy (1.8–2.0 Gy per fraction) in intermediate and high-risk prostate cancer patients. The increasingly higher doses also intensifies toxicities to the organs at risk (OARs) which can be partially overcome by using advanced planning techniques such as IMRT or a concomitant boost approach (6–14). However, dose escalation has not typically been performed in conjunction with pelvic nodal radiation. The pelvic dose bath may make it difficult to safely dose escalate the prostate gland while respecting normal tissue constraints to the OARs.

Recent literature suggests that prostate cancer may be different than other malignancies in terms of its slow proliferation rate. Labeling indexes can be extraordinarily low, with most reports suggesting levels below 1%, and longer potential doubling times with a median T_pot _value of 40 days (range 15 to 170) [15]. Traditionally, an alpha:beta ratio of 10 Gy is used to calculate the biologically equivalent dose (BED) for acute toxicity and tumour response. Current studies are predicting an alpha:beta ratio of 1.5 Gy (range 0.8–2.2) for prostate carcinoma, below the classic alpha:beta ratio of 3 to 4 Gy for rectal late radiation effects [16-22]. This gives a potential therapeutic advantage for hypofractionated RT schedules over conventional fractionation by escalating the biologically equivalent dose in a shorter period of treatment time with better tumour control and reduced rectal toxicity [18,23-25]. Proposed biologically equivalent hypofractionated treatment schedules for prostate cancer have been suggested in the literature [18-20,24].

The aim of this comparative dosimetric analysis is to evaluate two pelvic treatment paradigms of either pelvic vessel contouring plus margin expansion (pelvic vessel targeting paradigm) or full pelvic content treatment excluding identified critical structures (normal tissue avoidance paradigm) in the setting of hypofractionated treatment of high-risk prostate cancer. Helical tomotherapy will be used as the dynamic intensity modulated radiation therapy solution for both treatment solutions. 3DCRT plans will be used for control comparisons.

## Methods and materials

### Patients and target/normal tissue contours

**Figure 1:**
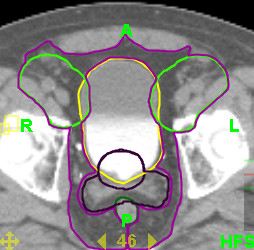
Example dosimetric volumes used for this study: Target – Prostate and Prostate/Seminal Vesicles PTVs, Nodal Target, Pelvic Target; Normal Tissue – Bladder and Rectum.

A sample of ten patients were scanned on a helical CT scanner (Phillips 5000) with 3 mm slice thickness with comfortably full bladder and no bowel preparation prior to simulation. The prostate and seminal vesicles were identified and contoured on each patient (by JY) and reviewed by two clinicians (GR, GB) in order to generate consensus-based contours. The PTV1 was defined as prostate + 7.5 mm (Figure 1). The nodal target was defined by a method proposed by Shih et al [26]. The distal common iliac (2 cm superior to the common iliac bifurcation), internal iliac (4 cm distal to bifurcation of the common iliac), and external iliac vessels (to the top of the superior pubic symphysis) were outlined from L5-S1 to the top of the symphysis pubis.

The conformal pelvic vessel targeting paradigm was assessed by generating a lymph node planning target volume which was defined by a 20 mm radial expansion of the contoured vessels and tailored to respect the muscle and bony pelvis normal tissue boundaries up to 10 mm. Therefore the final PTV_cpvt _for conformal pelvic vessel targeting included both PTV1 and the lymph node planning target volume (Figure 1). The conformal pelvic normal tissue avoidance paradigm was assessed by generating a pelvic soft tissue target which was defined as the pelvic soft tissue volume within a standard four field box. This volume exists between the previously defined lymph node planning target volume, respecting the normal tissue boundaries of muscle and bone, and subtracting out all other identified targets such as small bowel, bladder, rectum, and femora. Therefore, the PTV_cnta _for conformal normal tissue avoidance was the PTV1 + lymph node planning target volume + pelvic soft tissue target (Figure 1). In both planning cases, the simultaneous in-field boost (SIB) prostate boost volume would be PTV1.

Rectum, bladder and femoral heads were outlined using the guidelines provided by the RTOG P-0126 protocol. Specifically, the entire outer wall of the bladder is contoured, the rectum is contoured from the anus (at the level of the ischial tuberostities) for a length of 15 cm or to where the rectosigmoid flexure is identified. Femurs include the femoral head and extend inferiorly to the level of the ischial tuberosity. Small bowel was contoured in all slices where the nodal target or pelvic target was identified. All critical structures were contoured as a single volumetric structure and considered to be solid organs for dosimetric calculations. A prescription dose of 68 Gy was prescribed to 95% of the PTV1 in 25 fractions. PTV_cpvt _and PTV_cnta _were prescribed 45 Gy in the same 25 fractions for both the conformal pelvic vessel targeting and conformal normal tissue avoidance strategies, respectively.

### Helical tomotherapy planning

The dynamic IMRT solution chosen for this dosimetric feasibility study was helical tomotherapy (TomoTherapy Inc., Madison, WI, USA). CT datasets and structures were transferred to the TomoTherapy planning workstation using the DICOM RT protocol. The TomoTherapy station re-sampled the CT datasets in 256 × 256 voxels with the slice thickness re-sampled to the smallest slice separation in the original CT dataset. The planning system used an inverse treatment planning process based on iterative least squares minimization of an objective function [27]. Initial precedence, importance, and penalty factors were set (Table 1) to obtain a preliminary helical tomotherapy plan. Subsequent optimization was based on an assessment of target and OAR dose-volume parameters that have not been achieved and altering the penalty factors associated with the target/OAR to drive the plan optimization. The solutions must have resulted in deliverable treatment and could not exceed 30 minutes for total treatment delivery. The dose was calculated using a superposition/convolution approach [28,29]. Helical delivery is emulated in calculating 51 projections per rotation and the dose calculation uses a total of 24 different angles for the dose spread array of the incident 6 MV beam. The optimization algorithm is deterministic which allowed for the direct comparison of different strategies. A standardized class solution with a fan beam width of 11 mm, a pitch of 0.5, modulation factor of 3 and a dose calculation grid of approximately 4 × 4 × 3 mm^3 ^was used [30].

**Table 1:** Tumor and Normal Tissue Initial Tomotherapy Plan Optimization Parameters

**Tumor Constraints**							
Conformal Pelvic Vessel Targeting							

Structure	Importance	Max Dose (Gy)	Max Dose Penalty	DVH Volume (%)	DVH Dose (Gy)	Minimum Dose (Gy)	Minimum Dose Penalty

PTV1	10	68	10	95	68	68	33
Rt Iliac Nodal Volume	10	68	1	95	45	45	10
Lt Iliac Nodal Volume	10	68	1	95	45	45	10

PTV1 = prostate + 7.5 mm							

Conformal Normal Tissue Avoidance							

Structure	Importance	Max Dose (Gy)	Max Dose Penalty	DVH Volume (%)	DVH Dose (Gy)	Minimum Dose (Gy)	Minimum Dose Penalty

PTV1	10	68	10	95	68	68	33
PTV_cnta_	10	68	10	95	45	45	10

PTV_cnta _= PTV1 + lymph node planning target volume + pelvic soft tissue target							

**Sensitive Structure Constraints**							

Structure	Importance	Max Dose (Gy)	Max Dose Penalty	DVH Volume (%)	DVH Dose (Gy)	DVH Penalty	

Rectum	1	68	1	40	30	1	
Bladder	1	68	1	40	30	1	
Lt Femur	1	45	1	50	20	1	
Rt Femur	1	45	1	50	20	1	
Small Bowel	1	40	1	1	38	1	

Field Width = 5.0 cm Pitch = 0.286 Planning Modulation Factor = 4.0							

#### Three-dimensional conventional planning

3DCRT plans with 18 MV photons were generated using a commercial treatment planning system, Pinnacle DCM7.6c (Philips, Amsterdam, The Netherlands). The plans that were developed used a four-field technique to treat the pelvis and will serve as the control arm for this dosimetric study. For the anterior/posterior fields the superior border was at L5-S1, lateral borders 2 cm lateral to the widest point of the bony pelvic inlet, and inferior border 1.5 cm below the prostate on CT images. For the lateral fields, the anterior border was the anterior surface of the pubic symphysis, posterior border was the middle of the sacrum, including at least a posterior 0.75 cm margin on the prostate and seminal vesicle. Superior and inferior margins were identical to the anterior/posterior fields. The simultaneous in-field (SIB) prostate boost was treated with a 6 field coplanar technique targeting the prostate and proximal seminal vesicle with 1 cm margin. Shielding using 120 multi-leaf collimation (MLC) was used to shape the fields.

### Statistical methodology

The dIMRT/HT plans were compared to each other and the 3DCRT in terms of *a priori *defined target and normal tissue dose volume histogram (DVH) and dose metric outcome characteristics (Table 2). The *a priori *null hypothesis, for all comparisons, was that the mean values of DVH parameters/metrics between all three paradigms were not different. The alternate hypothesis was that the mean DVH parameters/metrics between all three paradigms were different. All main comparisons were performed using repeated measures analysis of variance (ANOVA). All two-way (between any two paradigms) post-hoc comparisons were performed using paired Bonferroni adjusted Student's t-tests.

**Table 2:** Target and Normal Tissue Dose Metrics Utilized in Study

Volumes of Interest		Dose Metrics
**Targets**	PTV1 Lt and Rt lymph node planning volumes Pelvic soft tissue target volume	D_99_, D_95_, D_5_, D_1_, D_99_-D_1_, D_95_-D_5_
**OARs**	Rectum, Bladder	D_50_, D_35_, D_25_, D_15_
	Small Bowel	D_5_, D_1_
	Femoral Head	D_15_

PTV = Planning Target Volume; OAR = Organ at Risk, D = Dose

## Results

### Target structures

The ten CT planning studies represent a wide range of potential target and normal tissue volumes (Table 3). All three planning strategies were able to cover 95% of the PTV1 with the prescription dose. Comparing one planning process to the other, there are statistically significant differences in the delivery of dose to this PTV1 (Table 4). When assessing dose homogeneity as defined as both D99-D1 and D95-D5, the conformal normal tissue avoidance solution showed the most homogeneous dose distribution compared to the other two strategies. 3DCRT delivered a higher absolute dose to the nodal target volume at all dose points (Table 5). However, both dIMRT/HT plans were able to deliver the prescription dose to the nodal target while being significantly more homogeneous. The pelvic soft tissue target volume looks specifically at the soft tissues within the pelvic field that excludes the nodal target and the organs at risk (Table 6). Given the highly conformal nature of tomotherapy, the conformal pelvic vessel targeting approach delivered a significantly lower dose to the pelvic soft tissues, as they were not specifically targeted. As expected, the 3DCRT and conformal normal tissue avoidance strategies delivered the highest dose to the pelvic soft tissue target volume. The conformal normal tissue avoidance technique had better homogeneity of dose compared to the 3DCRT control due to the IMRT delivery of helical tomotherapy.

**Table 3:** Volume Characteristics of 10 Patient CT datasets.

Structure	Mean (cm^3^)	SD (cm^3^)	Range (cc)
Prostate	56.86	36.12	30–144.7
Seminal Vesicle	14.82	6.42	3.77–23.5
Bladder	157.65	88.51	63.7–293.4
Small Bowel	244.78	130.89	43.6–496.88
Rectum	102.42	53.51	49.78–227.4
Pelvic Soft Tissues	720.32	241.20	460–1112.6
Left Nodal Target	426.8	61.04	349.33–510.29
Right Nodal Target	419.47	71.07	306.15–538.33
Left Femoral Head	181.38	26.75	151.28–226.66
Right Femoral Head	184.68	28.39	145.6–230.76

SD = Standard Deviation

**Table 4:** Dose Volume Metrics of the PTV1

	3DCRT	Targeting	Avoidance	ANOVA	3DCRT – Targeting	3DCRT – Avoidance	Targeting – Avoidance
D99%	65.18 ± 1.68	65.60 ± 0.59	66.91 ± 0.59	P < 0.001	P < 0.001	P < 0.001	P = 0.001
D95%	68.03 ± 0.97	68.04 ± 0.16	68.06 ± 0.08	P < 0.001	P < 0.001	P < 0.001	NS
D5%	72.15 ± 0.46	71.69 ± 0.35	70.22 ± 1.10	P < 0.001	P < 0.001	P = 0.004	P = 0.005
D1%	72.30 ± 0.47	72.35 ± 0.49	70.84 ± 1.09	P < 0.001	P < 0.001	P < 0.001	P = 0.002
D99%-D1%	-7.12 ± 1.63	-6.75 ± 0.95	-3.93 ± 1.63	P < 0.001	NS	P = 0.002	P = 0.001
D95%-D5%	-4.12 ± 0.92	-3.65 ± 0.34	-2.16 ± 1.09	P < 0.001	NS	P < 0.001	P = 0.002

3DCRT = Three Dimensional Conformal Radiation Therapy; Targeting = Conformal Pelvic Vessel Targeting; Avoidance = Conformal Normal Tissue Avoidance; ANOVA = Repeated Measures Analysis of Variance

**Table 5:** Dose Volume Metrics of the Nodal Target Volumes

	3DCRT	Targeting	Avoidance	ANOVA	3DCRT – Targeting	3DCRT – Avoidance	Targeting – Avoidance
Left Nodal Target Volume							
D99%	47.11 ± 0.61	43.49 ± 1.44	44.02 ± 0.86	P = 0.045	NS	NS	NS
D95%	48.36 ± 0.50	45.41 ± 0.76	45.23 ± 0.50	P = 0.02	NS	P = 0.005	NS
D5%	61.13 ± 2.80	49.02 ± 0.90	49.61 ± 1.40	P < 0.001	P < 0.001	P < 0.001	NS
D1%	63.57 ± 3.72	50.74 ± 2.22	51.99 ± 2.87	P < 0.001	P < 0.001	P < 0.001	NS
D99%-D1%	-16.45 ± 4.09	-7.25 ± 3.18	-8.42 ± 2.31	P < 0.001	P < 0.001	P < 0.001	NS
D95%-D5%	-12.77 ± 3.08	-3.61 ± 1.31	-4.60 ± 1.19	P < 0.001	P < 0.001	P < 0.001	NS
Right Nodal Target Volume							
D99%	46.97 ± 0.59	43.29 ± 2.00	43.39 ± 0.74	P = 0.04	NS	P < 0.001	NS
D95%	48.22 ± 0.58	45.21 ± 1.01	45.00 ± 0.49	P = 0.03	NS	P = 0.005	NS
D5%	61.12 ± 2.25	49.11 ± 0.98	49.65 ± 1.50	P < 0.001	P < 0.001	P < 0.001	NS
D1%	63.90 ± 3.39	51.08 ± 3.34	52.11 ± 3.40	P < 0.001	P < 0.001	P < 0.001	NS
D99%-D1%	-16.94 ± 3.48	-7.79 ± 4.66	-9.27 ± 3.37	P < 0.001	P < 0.001	P < 0.001	NS
D95%-D5%	-12.91 ± 2.46	-3.90 ± 1.68	-4.91 ± 1.34	P < 0.001	P < 0.001	P < 0.001	NS

**Table 6:** Dose Volume Metrics for the Pelvic Soft Tissue Target

	3DCRT	Targeting	Avoidance	ANOVA	3DCRT – Targeting	3DCRT – Avoidance	Targeting – Avoidance
D99%	47.11 ± 0.48	19.58 ± 3.00	41.35 ± 1.30	P < 0.001	P < 0.001	P < 0.001	P < 0.001
D95%	48.32 ± 0.41	27.30 ± 3.00	44.28 ± 1.18	P < 0.001	P < 0.001	P = 0.007	P < 0.001
D5%	62.09 ± 3.14	49.71 ± 1.84	53.49 ± 3.12	P < 0.001	P < 0.001	P = 0.001	P = 0.001
D1%	70.70 ± 1.35	56.53 ± 5.45	58.40 ± 4.44	P < 0.001	P < 0.001	P < 0.001	P = 0.047
D99%-D1%	-23.58 ± 1.19	-36.95 ± 6.43	-17.05 ± 3.93	P < 0.001	P < 0.001	P = 0.002	P < 0.001
D95%-D5%	-13.77 ± 3.19	-22.41 ± 3.65	-9.21 ± 2.17	P < 0.001	P < 0.001	P = 0.002	P < 0.001

### Organs at risk

DVH characteristics were compared for the rectum, bladder, femoral heads, and small bowel (Table 7). The 3DCRT plan generated the highest dose to all the organs at risk. The dIMRT/HT techniques were both able to significantly spare the critical structures better than the non-conformal control. Within the two dIMRT/HT approaches, conformal pelvic vessel targeting delivered a lower dose at most dose points in comparison to conformal normal tissue avoidance.

**Table 7:** Dose Volume Metrics for the Organs at Risk

	3DCRT	Targeting	Avoidance	ANOVA	3DCRT – Targeting	3DCRT – Avoidance	Targeting – Avoidance
Rectum							
D15%	67.68 ± 2.60	59.81 ± 6.44	62.45 ± 4.46	P = 0.004	P = 0.033	NS	NS
D25%	63.57 ± 3.62	52.14 ± 5.16	55.27 ± 4.81	P < 0.001	P < 0.001	P < 0.001	NS
D35%	59.39 ± 3.97	46.97 ± 4.58	50.00 ± 4.08	P < 0.001	P < 0.001	P < 0.001	P = 0.04
D50%	54.08 ± 3.22	39.99 ± 5.25	43.76 ± 3.18	P < 0.001	P < 0.001	P < 0.001	P = 0.034
Bladder							
D15%	69.14 ± 1.99	62.89 ± 4.61	63.75 ± 3.06	P = 0.004	P = 0.05	P = 0.02	NS
D25%	64.60 ± 5.37	56.01 ± 5.51	57.79 ± 4.43	P < 0.001	P < 0.001	P = 0.001	NS
D35%	60.54 ± 6.44	49.95 ± 6.51	52.61 ± 5.55	P < 0.001	P = 0.001	P = 0.001	P = 0.034
D50%	56.69 ± 5.34	42.40 ± 7.56	44.97 ± 4.83	P < 0.001	P < 0.001	P = 0.002	NS
Femora							
LFHD15%	62.84 ± 3.70	40.23 ± 3.64	42.29 ± 1.07	P < 0.001	P = 0.004	P < 0.001	NS
RFHD15%	62.55 ± 3.33	43.08 ± 10.03	41.83 ± 1.34	P < 0.001	P < 0.001	P < 0.001	NS
Small Bowel							
D5%	53.47 ± 1.63	42.50 ± 2.02	46.16 ± 2.48	P < 0.001	P < 0.001	P = 0.005	P = 0.002
D1%	54.31 ± 1.57	45.75 ± 2.46	50.00 ± 3.08	P < 0.001	P = 0.001	NS	P = 0.003

LFHD = Left femoral head dose; RFHD = Right femoral head dose

#### Dosimetric summary

When compared to conformal pelvic vessel targeting, conformal normal tissue avoidance delivered more homogenous PTV delivery (2/2 t-test comparisons; P < 0.001, Table 4), similar nodal coverage (8/8 t-test comparisons; p = ns, Table 5), higher and more homogenous pelvic tissue dose (6/6 t-test comparisons; P < 0.03, Table 6), at the cost of slightly higher critical structure dose (D_dose_, 1–3 Gy over 5/10 dose points; P < 0.03, Table 7). The dIMRT/HT approaches were superior to 3DCRT in sparing organs at risk (22/24 t-test comparisons; P < 0.05, Table 7).

## Discussion

Intensity modulated radiation therapy (IMRT) uses an advanced planning technique that creates complex dose distributions that can deliver a radical dose of radiation to the prostate gland and treat the pelvic nodes at risk, while reducing the irradiated volume of small bowel and rectum [31]. In addition, IMRT can be used to deliver dose to the primary prostate volume while simultaneously treating the regional lymph nodes at risk to a lower dose in a single phase. This strategy, called an SIB technique has many clinical, dosimetric, and economic advantages and has been incorporated into several different anatomic sites [32-39]. Integrating the whole pelvis and prostate boost into the plan optimization from the outset may, in theory, improve the likelihood that the resulting solution will be able to meet the constraints for safe prostate dose escalation in the setting of whole pelvis treatment. By using a SIB scheme, the prostate gland can be irradiated with a radical hypofractionated dose schedule while the pelvic nodes would receive a conventionally fractionated traditional microscopic dose [40].

Using IMRT, a conformal pelvic vessel targeting solution can be acheived to treat the prostate gland while also treating the pelvic node bearing regions if the physician can reliability identify these treatment volumes. In the area of head and neck radiotherapy, standardized and reliable anatomic maps for contouring lymph node regions are available [41,42]. However, no consensus exists for a standardized identification of pelvic lymph node anatomy exists. Currently, contouring of the pelvic vessels has been used as a surrogate for pelvic nodal regions and used to generate clinical target volumes. This is usually done by adding a 1.5 to 2 cm margin around the vessel itself to approximate the region of the perivascular lymph nodes [26]. Several potential difficulties exist with this conformal pelvic vessel targeting approach. Firstly, there is uncertainty as to the optimal margin of normal tissue around the vessels to adequately cover the lymph node bearing regions. Secondly, there can be difficulty in the tracking and visualizing of the internal iliac vasculature. Finally, there is an inability to target smaller lymphatic vessels and lymph node regions "in transit" to the larger nodal stations along the visible vessels.

An alternate strategy proposed in relation to this study is conformal normal tissue avoidance. In this solution, the goal is to identify the organs at risk (bladder, small bowel, rectum, and femoral heads) and subtract them from the pelvic target volume. The remaining volume is identified as the target for regional nodal irradiation, which contains the soft tissues of the pelvis (corresponding to the pelvis at risk that would be treated by a standard non conformal pelvic radiation field). Inversely or forward planned optimization can then be designed to treat the pelvic soft tissue target volume to a microscopic dose while limiting dose to the identified critical structures and dose escalating the prostate gland. This approach carries the advantage that the critical structures are typically easier to identify as avoidance volumes rather than the nodal target regions (which rely on vessels as a surrogate marker). The conformal normal tissue avoidance strategy would also allow treatment of smaller lymphatic vessels and lymph nodes within the pelvic soft tissues with a lower risk of under-treating important nodal regions. Problems with this approach include a modest increase in dose to the organs at risk compared to the conformal pelvic vessel targeting approach and the effect of inter-fraction organ movement. Multiple CT simulations or daily image guidance with adaptive therapy may be required to clinically implement a pelvic conformal avoidance strategy. However it is important to note that doses to the OAR's compare favorably to the calculated and expected doses in conjunction with 3DCRT four-field pelvic radiation.

In this paper, we attempt to incorporate hypofractionation, dose escalation, and nodal basin irradiation within a single-phase dynamic IMRT helical tomotherapy (dIMRT/HT) solution. Two opposing strategies were studied, conformal pelvic vessel targeting and conformal normal tissue avoidance, using the unique capabilities of a TomoTherapy treatment planning and image-guidance and IMRT radiation delivery system. Even though both strategies differ in their approach to the nodal basin, both solutions delivered the prescribed dose to the prostate and vessel-defined node bearing regions. The major difference lies in the dose to the pelvic soft tissues that lie between the expanded nodal target volume and the organs at risk. Conformal pelvic vessel targeting does not specifically address these tissues and subsequently the planning system algorithm cannot use this information in developing a dosimetric plan. The dose is driven into the defined nodal target and this area essentially becomes a buffer zone where a dose gradient exists between the vessel targets and the organs at risk. As such, the planned dose is significantly less than in the conformal normal tissue avoidance paradigm where this area is specifically defined as a target. The planning system optimizes based on the importance, precedence, and penalty factors to deliver dose to the pelvic soft tissue target with no such buffer zone between it and the organs at risk. Therefore, the conformal normal tissue avoidance technique was able to deliver the microscopic dose to the pelvic tissues while having the benefit of not having to define a nodal target region based on potentially ill-defined pelvic vasculature. In addition, the concern of geometric miss associated with many conformal treatments (due to issues such as motion of the target) are minimized.

Because conformal normal tissue avoidance targets all the tissue within the pelvis aside from the organs at risk; it necessarily delivers a higher dose to the organs at risk when compared to conformal pelvic vessel targeting unless they are specifically excluded as a critical structure. We can see this from the data in table seven, which shows statistically significant higher doses to these organs at 8/12 dose points. The absolute differences were about 1–4 Gy over the entire course of treatment, which may be of limited or no clinical significance in terms of differences in possible late toxicity. This potential cost to the normal tissues is necessary to deliver the dose described to the rest of the pelvis. The clinical impact of this difference in terms of acute and late effects is currently unknown.

Unfortunately, there are no defined dose limits to OARs in the setting of hypofractionated treatment of the pelvis. However, using the linear quadratic concept to calculate biological effective doses of different fractionation protocols we can compare our planned doses with the dose limits given for a large RTOG dose escalation trial (Table 8). The regimens proposed here for hypofractionated dose escalated treatment of the prostate gland is based on currently available data. The reliability of each radiobiologic model will limit our BED. However, even if the α/β of prostate is 3 instead of 1.5, our planned dose will still deliver a BED (2 Gy) of 78 Gy. We can see that the planned doses using both dIMRT/HT strategies are within the dose constraints given by RTOG P0126. Even so, the impact on normal tissues of a hypofractionated protocol where the overall treatment time is significantly less will need to be defined in current and future clinical trials. In Canada, a clinical trial is underway evaluating linac based IMRT and helical tomotherapy, clinically assessing a dose regimen of 68 Gy in 25 fractions to the prostate while simultaneously delivering 45 Gy in 25 fractions to pelvic tissues.

**Table 8:** Comparing Dose to Bladder and Rectum to Dose Constraints from RTOG P0126 Protocol

	D15% (Gy)	D25% (Gy)	D35% (Gy)	D50% (Gy)
RTOG Rectum	75	70	65	60
RTOG Rectum over 25 fractions	63.63	59.66	55.68	51.67
Targeting	59.81	52.14	46.97	39.99
Avoidance	62.45	55.27	50.00	43.76
RTOG Bladder	80	75	70	65
RTOG Bladder over 25 fractions	67.58	63.63	59.66	55.68
Targeting	62.89	56.01	49.95	42.40
Avoidance	63.75	57.79	52.61	44.97

RTOG = Radiation Therapy Oncology Group

The effects of normal tissue movement are not taken into account here. While the nature of daily MVCT localization of the prostate is an inherent benefit to tomotherapy treatment, it currently does not take into account the daily movement of normal tissues. Ideally, a planning system powerful enough to develop a solution daily within the time constraints of a busy treatment facility would be the ultimate solution. However, as an interim step the concept of adding a margin for tissue movement can also be used as suggested by the ICRU. We expect that planning with a more realistic OAR volume will result in a plan that would lie between the extremes of conformal pelvic vessel targeting and conformal normal tissue avoidance presented here. Clinical investigations into the appropriate definition of the nodal targets are also under evaluation. For instance, studies into ultra-small super-paramagnetic iron oxide particles, known generically as ferumoxtran-10, have been successfully evaluated for detection of sentinel lymph nodes in various clinical trials [43-45]. Anatomic nodal information derived from these studies may better define the regions at risk within the pelvis to identify to our treatment planning systems and subsequently drive the planning system optimization to better cover the intended targets and to continue to spare the OAR's.

The techniques developed here extend beyond the treatment of prostate cancer. Similar approaches can be used in other disease sites within the pelvis (cervix, endometrium, etc). Also, the concepts of conformal normal tissue avoidance can be generalized to wherever there is a concern over uncertainties regarding pelvic nodal target delineation and nearby organs at risk. This technical dosimetric feasibility study offers evidence that conformal avoidance, as an advanced treatment planning strategy, is a potential solution to deliver highly conformal pelvic radiation in the setting of nodal location uncertainty due to incomplete nodal mapping or abherent nodal drainage.

## Conclusion

Therefore this research study has demonstrated that dIMRT/HT nodal and pelvic targeting is superior to 3DCRT in dose delivery and critical structure sparing in the setting of hypofractionation for high-risk prostate cancer. This technical dosimetric feasibility study offers evidence that conformal avoidance, as an advanced treatment planning strategy, is a potential solution to deliver highly conformal pelvic radiation in the setting of nodal location uncertainty due to incomplete nodal mapping or complex nodal drainage.

## Competing interests

The author(s) declare that they have no competing interests.

## Authors' contributions

All authors have read and approved the final manuscript. Specifically, JY completed all contours, supervised treatment planning, performed interpretation of statistical analysis, and drafted/approved the manuscript. GR was responsible for the initial research idea, supervision of the project, statistical analysis, assisted in the preparation and approval of the manuscript. TC and KT performed treatment planned, assisted in the preparation and approval of the final manuscript. SY, ML, DD, and GB co-supervised the project, assisted in the interpretation of the statistical analysis, and assisted in the preparation and approval of the manuscript.

## References

[B1] Canadian Cancer Society/National Cancer Institute of Canada (2006). Canadian Cancer Statistics 2006. Toronto, Canada.

[B2] Lukka H, Warde P, Pickles T, Morton G, Brundage M, Souhami L, Canadian GU Radiation Oncologist Group (2001). Controversies in prostate cancer radiotherapy: Consensus development. Can J Urol.

[B3] Roach M, DeSilvio M, Lawton C, Uhl V, Machtay M, Seider MJ, Rotman M, Jones C, Asbell SO, Valicenti RK, Han S, Thomas CR, Shipley WS, Radiation Therapy Oncology Group 9413 (2003). Phase III trial comparing whole-pelvic versus prostate-only radiotherapy and neoadjuvant versus adjuvant combined androgen suppression: Radiation therapy oncology group 9413. J Clin Oncol.

[B4] Bolla M, Collette L, Blank L, Warde P, Dubois JB, Mirimanoff RO, Storme G, Bernier J, Kuten A, Sternberg C, Mattelaer J, Lopez Torecilla J, Pfeffer JR, Lino Cutajar C, Zurlo A, Pierart M (2002). Long-term results with immediate androgen suppression and external irradiation in patients with locally advanced prostate cancer (an EORTC study): A phase III randomised trial. Lancet.

[B5] Hanks GE, Pajak TF, Porter A, Grignon D, Brereton H, Venkatesan V, Horwitz EM, Lawton C, Rosenthal SA, Sandler HM, Shipley WU, Radiation Therapy Oncology Group (2003). Phase III trial of long-term adjuvant androgen deprivation after neoadjuvant hormonal cytoreduction and radiotherapy in locally advanced carcinoma of the prostate: The radiation therapy oncology group protocol 92–02. J Clin Oncol.

[B6] Pollack A, Zagars GK, Starkschall G, Antolak JA, Lee JJ, Huang E, von Eschenbach AC, Kuban DA, Rosen I (2002). Prostate cancer radiation dose response: Results of the M. D. anderson phase III randomized trial. Int J Radiat Oncol Biol Phys.

[B7] Valicenti RK, Winter K, Cox JD, Sandler HM, Bosch W, Vijayakumar S, Michalski J, Purdy J (2003). RTOG 94-06: Is the addition of neoadjuvant hormonal therapy to dose-escalated 3D conformal radiation therapy for prostate cancer associated with treatment toxicity?. Int J Radiat Oncol Biol Phys.

[B8] Bos LJ, Damen EM, de Boer RW, Mijnheer BJ, McShan DL, Fraass BA, Kessler ML, Lebesque JV (2002). Reduction of rectal dose by integration of the boost in the large-field treatment plan for prostate irradiation. Int J Radiat Oncol Biol Phys.

[B9] Amer AM, Mott J, Mackay RI, Williams PC, Livsey J, Logue JP, Hendry JH (2003). Prediction of the benefits from dose-escalated hypofractionated intensity-modulated radiotherapy for prostate cancer. Int J Radiat Oncol Biol Phys.

[B10] Beckendorf V, Guerif S, Le Prise E, Cosset JM, Lefloch O, Chauvet B, Salem N, Chapet O, Bourdin S, Bachaud JM, Maingon P, Lagrange JL, Malissard L, Simon JM, Pommier P, Hay MH, Dubray B, Luporsi E, Bey P (2004). The GETUG 70 gy vs. 80 gy randomized trial for localized prostate cancer: Feasibility and acute toxicity. Int J Radiat Oncol Biol Phys.

[B11] Pollack A, Zagars GK, Smith LG, Lee JJ, von Eschenbach AC, Antolak JA, Starkschall G, Rosen I (2000). Preliminary results of a randomized radiotherapy dose-escalation study comparing 70 gy with 78 gy for prostate cancer. J Clin Oncol.

[B12] Hanks GE, Hanlon AL, Pinover WH, Horwitz EM, Schultheiss TE (1999). Survival advantage for prostate cancer patients treated with high-dose three-dimensional conformal radiotherapy. Cancer J Sci Am.

[B13] Hanks GE, Hanlon AL, Schultheiss TE, Pinover WH, Movsas B, Epstein BE, Hunt MA (1998). Dose escalation with 3D conformal treatment: Five year outcomes, treatment optimization, and future directions. Int J Radiat Oncol Biol Phys.

[B14] Zelefsky MJ, Leibel SA, Kutcher GJ, Fuks Z (1998). Three-dimensional conformal radiotherapy and dose escalation: Where do we stand?. Semin Radiat Oncol.

[B15] Haustermans KM, Hofland I, Van Poppel H, Oyen R, Van de Voorde W, Begg AC, Fowler JF (1997). Cell kinetic measurements in prostate cancer. Int J Radiat Oncol Biol Phys.

[B16] Wang JZ, Guerrero M, Li XA (2003). How low is the alpha/beta ratio for prostate cancer?. Int J Radiat Oncol Biol Phys.

[B17] Wang JZ, Li XA, Yu CX, DiBiase SJ (2003). The low alpha/beta ratio for prostate cancer: What does the clinical outcome of HDR brachytherapy tell us?. Int J Radiat Oncol Biol Phys.

[B18] Brenner DJ, Hall EJ (1999). Fractionation and protraction for radiotherapy of prostate carcinoma. Int J Radiat Oncol Biol Phys.

[B19] Fowler J, Chappell R, Ritter M (2001). Is alpha/beta for prostate tumors really low?. Int J Radiat Oncol Biol Phys.

[B20] Brenner DJ, Martinez AA, Edmundson GK, Mitchell C, Thames HD, Armour EP (2002). Direct evidence that prostate tumors show high sensitivity to fractionation (low alpha/beta ratio), similar to late-responding normal tissue. Int J Radiat Oncol Biol Phys.

[B21] King CR, Fowler JF (2001). A simple analytic derivation suggests that prostate cancer alpha/beta ratio is low. Int J Radiat Oncol Biol Phys.

[B22] Kal HB, Van Gellekom MP (2003). How low is the alpha/beta ratio for prostate cancer?. Int J Radiat Oncol Biol Phys.

[B23] Brenner DJ (2000). Toward optimal external-beam fractionation for prostate cancer. Int J Radiat Oncol Biol Phys.

[B24] Fowler JF, Ritter MA, Chappell RJ, Brenner DJ (2003). What hypofractionated protocols should be tested for prostate cancer?. Int J Radiat Oncol Biol Phys.

[B25] Brenner DJ (2003). Hypofractionation for prostate cancer radiotherapy – what are the issues?. Int J Radiat Oncol Biol Phys.

[B26] Shih HA, Harisinghani M, Zietman AL, Wolfgang JA, Saksena M, Weissleder R (2005). Mapping of nodal disease in locally advanced prostate cancer: rethinking the clinical target volume for pelvic nodal irradiation based on vascular rather than bony anatomy. Int J Radiat Oncol Biol Phys.

[B27] Shepard DM, Olivera GH, Reckwerdt PJ, Mackie TR (2000). Iterative approaches to dose optimization in tomotherapy. Phys Med Biol.

[B28] Papanikolaou N, Mackie TR, Meger-Wells C, Gehring M, Reckwerdt P (1993). Investigation of the convolution method for polyenergetic spectra. Med Phys.

[B29] Lu W, Olivera GH, Chen ML, Reckwerdt PJ, Mackie TR (2005). Accurate convolution/superposition for multi-resolution dose calculation using cumulative tabulated kernels. Phys Med Biol.

[B30] Grigorov G, Kron T, Wong E, Chen J, Sollazzo J, Rodrigues G (2003). Optimization of helical tomotherapy treatment plans for prostate cancer. Phys Med Biol.

[B31] Nutting CM, Convery DJ, Cosgrove VP, Rowbottom C, Padhani AR, Webb S, Dearnaley DP (2000). Reduction of small and large bowel irradiation using an optimized intensity-modulated pelvic radiotherapy technique in patients with prostate cancer. Int J Radiat Oncol Biol Phys.

[B32] Lebesque JV, Keus RB (1991). The simultaneous boost technique: The concept of relative normalized total dose. Radiother Oncol.

[B33] Schuster-Uitterhoeve AL, Hulshof MC, Gonzalez Gonzalez D, Koolen M, Sminia P (1993). Feasibility of curative radiotherapy with a concomitant boost technique in 33 patients with non-small cell lung cancer (NSCLC). Radiother Oncol.

[B34] Heukelom S, Lanson JH, Mijnheer BJ (1994). Quality assurance of the simultaneous boost technique for prostatic cancer: Dosimetric aspects. Radiother Oncol.

[B35] Butler EB, Teh BS, Grant WH, Uhl BM, Kuppersmith RB, Chiu JK, Donovan DT, Woo SY (1999). Smart (simultaneous modulated accelerated radiation therapy) boost: A new accelerated fractionation schedule for the treatment of head and neck cancer with intensity modulated radiotherapy. Int J Radiat Oncol Biol Phys.

[B36] Wu Q, Manning M, Schmidt-Ullrich R, Mohan R (2000). The potential for sparing of parotids and escalation of biologically effective dose with intensity-modulated radiation treatments of head and neck cancers: A treatment design study. Int J Radiat Oncol Biol Phys.

[B37] Mohan R, Wu Q, Manning M, Schmidt-Ullrich R (2000). Radiobiological considerations in the design of fractionation strategies for intensity-modulated radiation therapy of head and neck cancers. Int J Radiat Oncol Biol Phys.

[B38] Bos LJ, Damen EM, de Boer RW, Mijnheer BJ, McShan DL, Fraass BA, Kessler ML, Lebesque JV (2002). Reduction of rectal dose by integration of the boost in the large-field treatment plan for prostate irradiation. Int J Radiat Oncol Biol Phys.

[B39] Mott JH, Livsey JE, Logue JP (2004). Development of a simultaneous boost IMRT class solution for a hypofractionated prostate cancer protocol. Br J Radiol.

[B40] Li XA, Wang JZ, Jursinic PA, Lawton CA, Wang D (2005). Dosimetric advantages of IMRT simultaneous integrated boost for high-risk prostate cancer. Int J Radiat Oncol Biol Phys.

[B41] Gregoire V, Levendag P, Ang KK, Bernier J, Braaksma M, Budach V, Chao C, Coche E, Cooper JS, Cosnard G, Eisbruch A, El-Sayed S, Emami B, Grau C, Hamoir M, Lee N, Maingon P, Muller K, Reychler H (2003). CT-based delineation of lymph node levels and related CTVs in the node-negative neck: DAHANCA, EORTC, GORTEC, NCIC, RTOG consensus guidelines. Radiother Oncol.

[B42] Levendag P, Braaksma M, Coche E, van Der Est H, Hamoir M, Muller K, Noever I, Nowak P, van Sörensen De Koste J, Grégoire V (2004). Rotterdam and brussels CT-based neck nodal delineation compared with the surgical levels as defined by the american academy of otolaryngology-head and neck surgery. Int J Radiat Oncol Biol Phys.

[B43] Harisinghani MG, Barentsz J, Hahn PF, Deserno WM, Tabatabaei S, van de Kaa CH, dela Rosette J, Weissleder R (2003). Noninvasive detection of clinically occult lymph-node metastases in prostate cancer. N Engl J Med.

[B44] Taupitz M, Hamm BK, Barentsz JO, Vock P, Roy C, Bellin MF (1999). Sinerem-enhanced MRI imaging compared to plain MR imaging in evaluating lymph node metastases from urologic and gynaecologic cancers [abstract]. Proceedings of the Radiological Society of North America, Chicago, IL.

[B45] Anzai Y, Piccoli CW, Outwater EK, Stanford W, Bluemke DA, Nurenberg P, Saini S, Maravilla KR, Feldman DE, Schmiedl UP, Brunberg JA, Francis IR, Harms SE, Som PM, Tempany CM (2003). Evaluation of neck and body metastases to nodes with ferumoxtran 10-enhanced MR imaging: phase III safety and efficacy study. Radiology.

